# Secondary Healing of Fingertip Amputations: Simple Wound Care Advice for Patients

**DOI:** 10.1097/GOX.0000000000004020

**Published:** 2022-01-18

**Authors:** Donald H. Lalonde, Jasmine Bouhtiauy

**Affiliations:** From the * Division of Plastic Surgery, Dalhousie University, Saint John, New Brunswick, Canada; † Université de Sherbrooke, Moncton, Canada.

Not all patients want or need surgery to help heal their amputated fingertip.^1–4^ The videos and words in this article show patients how to look after amputated fingertips, with Vaseline, Coban tape, and running water over the wound daily at shower time. This simple inexpensive wound care plan is easy to follow until the wound heals without surgery at 4–8 weeks.

## HOW THE WOUND HEALS WITH CONSTANT VASELINE COVERAGE

Skin for coverage, fat for padding, nerves for feeling, and blood vessels for warmth are pulled over the exposed fat, bone, tendon, or joint by wound contracture as the defect closes by shrinking.^5^ Exposed red raw tissues (even the bone) will not die if they never dry. When raw tissues are drying, they start crying (pain) because they are dying. Do not let them dry. Thickly applied Vaseline keeps in the moisture and is replaced every day after the shower rinses away the old Vaseline and debris. Each day of healing, the pink healed area closes in over the shrinking red raw area. Stop Vaseline dressings when the wound is all pink with no more red. Red is raw. Pink is healed.

## COBAN IS THE ONLY BANDAGE

Sterile dressings are not required. Coban is a clean bandage that sticks to itself and keeps the Vaseline on the wound, so it does not dry. Other clean bandages such as a washable silicone finger cot also work. (See Video 1 [online], which displays showing a patient how to apply Vaseline and Coban to his amputated fingertip so it does not hurt.) (See Video 2 [online], which displays how to heal faster with pain guided healing) (See Video 3 [online], which displays tips and tricks for faster healing) (See Video 4 [online], which displays a time lapse photograph of wound healing after seeing the doctor only once for the initial consultation.)

**Video 1.** Reducing pain using Vaseline. Video 1 from “Secondary Healing of Fingertip Amputations: Simple Wound Care Advice to Patients.” This video displays showing a patient how to apply Vaseline and Coban to his amputated fingertip so it does not hurt.

**Video 2.** Healing faster. Video 2 from “Secondary Healing of Fingertip Amputations: Simple Wound Care Advice to Patients.” This video displays how to heal faster with pain guided healing.

**Video 3.** Tips to healing faster. Video 3 from “Secondary Healing of Fingertip Amputations: Simple Wound Care Advice to Patients.” This video displays tips and tricks for faster healing. ”

**Video 4.** Healing timelapse. Video 4 from “Secondary Healing of Fingertip Amputations: Simple Wound Care Advice to Patients.” This video displays a timelapse of wound healing after seeing the doctor only once for the initial consultation.

## MANAGING THE VASELINE

Too much Vaseline is better than not enough. If there is not enough, the bandage will get stuck and will be painful to remove. After a week of dressings, there may be an odor from the fingertip when you take off the Coban. If this happens, gently remove some of the old, thick, smelly layer of Vaseline and debris with a cotton-tipped stick (Q-tip) to solve the problem.

## DAILY SHOWERING

After removing the Coban, get in the shower with a naked finger. Do not stick your amputated finger directly under the shower nozzle as that would hurt. Simply let water (with or without soap) run over the wound.

*Don’t worry about maceration.* Pink skin around the wound looks macerated. This is the wrinkled, wet look that fingertips get if we have been in the bathtub too long. This is not a problem at all. Maceration disappears quickly after the wound is healed and Vaseline is stopped.
